# Functional Characterization of OsCSN1 in the Agronomic Trait Control of Rice Seedlings Under Far-Red Light

**DOI:** 10.3390/ijms26020522

**Published:** 2025-01-09

**Authors:** Yanxi Liu, Hua Zeng, Yuqing Shang, Hexin Zhang, Tongtong Jiao, Le Yin, Jinyuan Yang, Miao Xu, Jingmei Lu, Ming Wu, Liquan Guo

**Affiliations:** 1 College of Life Sciences, Jilin Agricultural University, Changchun 130118, China; lyx309660408@163.com (Y.L.); 18670148531@163.com (H.Z.); s1964006605@163.com (Y.S.); zhx03101212@163.com (H.Z.); jtt1163933169@163.com (T.J.); lyjiau@126.com (L.Y.); y208374306@163.com (J.Y.); miaox@jlau.edu.cn (M.X.); 2 School of Life Sciences, Northeast Normal University, Changchun 130024, China; jingmlu@163.com

**Keywords:** rice seedlings, far-red light, COP9 signalosome, N-terminal of OsCSN1

## Abstract

The COP9 signalosome (CSN) is a highly conserved multi-subunit protein complex, with CSN1 being its largest and most conserved subunit. The N-terminal function of CSN1 plays a pivotal and intricate role in plant photomorphogenesis and seedling development. Moreover, CSN is essential for far-red light-mediated photomorphogenesis in seedlings, but the function of OsCSN1 in seedling growth and development under far-red light conditions has not been determined. This study investigates the function of OsCSN1 under far-red light through phenotypic analysis of wild type and *OsCSN1* mutant seedlings. Additionally, the effect of the N-terminal region of OsCSN1 on rice seedling growth and development was examined. The addition of exogenous hormone gibberellin (GA_3_) and gibberellin synthesis inhibitor paclobutrazol (PAC) resulted in notable changes in phenotypes and the expression of key proteins, including CUL4 and SLR1. The findings indicate that OsCSN1 functions as a positive regulator of plant height under far-red light and inhibits root elongation. Under far-red light, OsCSN1 integrates into the COP9 complex and regulates the nuclear localization of COP1. Through its interaction with CUL4 in the CULLIN-RING family, OsCSN1 facilitates the ubiquitin-mediated degradation of SLR1, thereby influencing the growth of rice seedlings. The regulatory function of OsCSN1 in seedling growth and development under far-red light predominantly relies on the 32 amino acids of its N-terminal region. The results of this study can provide new ideas for rice breeding and genetic improvement. Based on the study of key regulatory factors such as OsCSN1, new varieties that can make better use of far-red light signals can be cultivated to enhance crop adaptability and productivity.

## 1. Introduction

The COP9 signalosome (CSN) was initially found to be a negative regulator of photomorphogenesis in Arabidopsis [1]. Moreover, a mutant of the CSN exhibits a constitutive photomorphogenesis phenotype under dark conditions [2]. Biochemical studies in plant and animal systems have shown that the CSN is a highly conserved nucleoprotein complex [3,4,5], with its structural integrity being essential for its biological activity [6,7,8]. The CSN shares significant structural and amino acid sequence homology with the “lid” subcomplex of the 19S regulatory particle in the 26S proteasome [3,4,5]. This similarity suggests that the CSN’s regulatory functions may be closely linked to ubiquitin/26S proteasome system-mediated protein degradation. The CSN interacts with various CULLIN-RING E3 ubiquitin ligases (CRLs) to affect various developmental processes in plants [9]. The COP9 signaling complex (CSN), as a highly conserved protein complex, plays an important role in the growth and development of rice. The CSN regulates intracellular protein levels by regulating the ubiquitin–proteasome system (UPS) to affect protein degradation. The CSN is involved in the regulation of hormone signal transduction pathways and promotes rice growth and development.

CSN1 is the largest and most conserved subunit in the CSN complex, plays a critical role in complex assembly through its C-terminal PCI domain, and supports essential functions via its N-terminal region [10]. The N-terminal domain is vital for plant growth and development, where its deletion leads to seedling lethality, floral organ abnormalities, and photomorphogenesis defects. CSN1 interacts with other subunits to restore the NEDD8/RUB1 deconjugation activity of CULLINs in csn1 mutants of Arabidopsis [11]. Additionally, mutants such as *csn5a*-*1* and *csn1*-*10* show delayed germination and accumulation of germination inhibitors RGL2 and ABI5, highlighting the CSN’s regulatory role in seed germination [12]. Overall, the N-terminal domain of CSN1 is indispensable for photomorphogenesis, seedling development, gene expression regulation, subcellular localization, and enzymatic activity within the complex. Its integration into the CSN complex is mediated by the PCI domain [10].

Light is a critical regulator of plant growth and development, primarily through photoreceptor-mediated signaling pathways that control photomorphogenesis and coordinate various developmental processes [13,14]. Far-red light (720–750 nm) influences key physiological processes, including seed germination, photomorphogenesis, and flowering, with its signaling pathway having complex effects on different physiological responses [15].

Phytochrome is the main receptor for plants to sense light and is widely present in higher plants, existing in two interconvertible forms: the far-red light-absorbing form (Pfr, 730 nm) and the red light-absorbing form (Pr, 660 nm) [16,17]. Phytochrome A (phyA) specifically senses and responds to far-red light, playing a key role in the transition from skotomorphogenesis to photomorphogenesis under far-red-rich light conditions [16]. Under far-red light conditions, the expression of the PHYA gene in rice is upregulated and regulates the photomorphogenesis of rice [18]. Far-red light rapidly induces phyA translocation to the nucleus within minutes [19], where it is subsequently degraded upon light exposure [20]. Far-red light inhibits photomorphogenesis by promoting the activity of SPA1, a negative regulator, which collaborates with COP1 to form an E3 ubiquitin ligase complex. COP1 mediates the light-induced degradation of phyA through the ubiquitin/26S-proteasome pathway, suppressing photomorphogenesis by targeting phosphorylated phyA and the positive regulator HY5 for degradation [21,22,23,24]. Consequently, plants exhibit elongated hypocotyls and increased height under far-red light [21,25].

Plant hormones play a crucial role in regulating plant growth and development by stimulating various physiological processes. Gibberellins (GAs) promote cell division and elongation during seed germination and are involved in flower development and fruit ripening [26,27]. Conversely, paclobutrazol, a growth regulator, inhibits endogenous gibberellin biosynthesis, thereby enhancing plant stress resistance and increasing crop yield [28]. DELLA proteins act as negative regulators of the GA signaling pathway [29]. During seed germination, DELLA proteins function as repressors, with GAs alleviating this repression by inhibiting DELLA protein activity to regulate germination and stem elongation [30]. In rice, SLR1, the sole DELLA protein, plays a central role in the GA signaling pathway, where its activity suppresses growth, leading to dwarfism during development [31].

Light is a critical environmental factor influencing rice seed germination and seedling development. Previous studies have demonstrated that OsCSN1 mediates the blue light-regulated inhibition of shoot growth in rice seedlings by affecting the degradation of SLR1, thereby reducing endogenous GA synthesis [32]. In contrast, OsCSN1 acts as a negative regulator of shoot elongation under red light. Red light promotes the degradation of SLR1 via neddylation and the ubiquitin/26S proteasome pathway, ultimately suppressing stem and coleoptile elongation [33]. These findings underscore the distinct effects of different light spectra on rice seedling growth and development.

This study focuses on OsCSN1 to investigate its role in regulating rice seedling growth under far-red light. The results reveal that far-red light, in combination with exogenous hormones such as GA_3_ and PAC, induces distinct phenotypes and alters protein and gene expression levels between wild type and *OsCSN1* mutants. Adjusting light and hormone treatments effectively promotes rice seedling growth and offers potential for enhancing agronomic traits in rice cultivation. Understanding the regulation mechanism of far-red light on rice gene expression is crucial for improving agronomic traits. Investigating the function of OsCSN1 may uncover its role in modulating gene expression, providing insights for the selection and cultivation of rice varieties adapted to diverse light conditions. Far-red light significantly influences the physiological and ecological characteristics of rice seedlings, impacting key agronomic traits such as yield, quality, and stress resistance. Therefore, elucidating these mechanisms will offer a scientific basis for optimizing rice cultivation practices and enhancing both yield and quality.

## 2. Results

### 2.1. OsCSN1 Is a Positive Regulator of Plant Height in Rice Seedlings Under Far-Red Light

**Figure 1:**
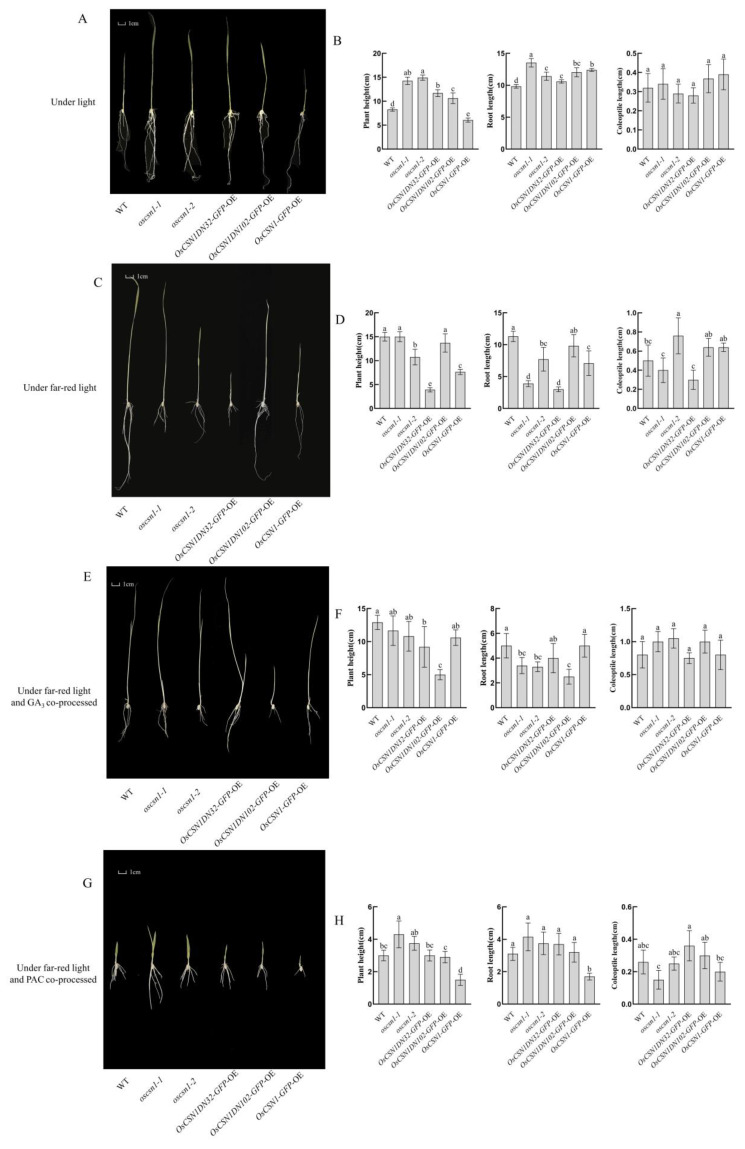
Phenotypic changes of wild type and *OsCSN1* mutants under natural light, far-red light, and exogenous GA_3_ and PAC hormone treatments. Data are mean ± SD (n = 5). Different letters represent significant differences between wild type and mutants. One-way analysis of variance (ANOVA) test followed by Duncan’s post hoc test (*p* < 0.05). (**A**) Phenotype of WT and *OsCSN1* mutants grown 9d under natural light. (**B**) Plant height, root length, and coleoptile length data graph of WT and *OsCSN1* mutants grown for 9d under natural light. (**C**) Phenotype of WT and *OsCSN1* mutants grown for 9d under far-red light. (**D**) Plant height, root length, and coleoptile length data graph of WT and *OsCSN1* mutants grown for 9d under far-red light. (**E**) Phenotype of WT and *OsCSN1* mutants grown for 9d under far-red light and GA_3_ co-processed. (**F**) Plant height, root length, and coleoptile length data graph of WT and *OsCSN1* mutants grown for 9d under far-red light and GA_3_ co-processed. (**G**) Phenotype of WT and *OsCSN1* mutants grown for 9d under far-red and PAC co-processed. (**H**) Plant height, root length, and coleoptile length data graph of WT and *OsCSN1* mutants grown for 9d under far-red and PAC co-processed.

Under far-red light, the height of the *oscsn1*-*2* and *OsCSN1DN32*-*GFP*-OE was significantly reduced (Figure 1C,D). Exogenous gibberellin (GA_3_) treatment notably increased the height of *OsCSN1DN32*-*GFP*-OE, while the height of *OsCSN1DN102*-*GFP*-OE remained inhibited compared to far-red light (Figure 1E,F). After the addition of the exogenous hormone PAC, the height of the wild type and mutants was lower than those under far-red light and the height of the *OsCSN1*-*GFP*-OE was significantly lower than that of the wild type (Figure 1G,H).

Compared with natural light, far-red light had certain effects on plant height (Figure 1A–D). Under far-red light, the *OsCSN1DN32*-*GFP*-OE exhibited a notable decrease in plant height, while *oscsn1*-*1* showed no significant change (Figure 1A–D). GA_3_ treatment under far-red light inhibited plant height in the knockout and N-terminal deletion mutants, but *OsCSN1*-*GFP*-OE treated with both far-red light and GA_3_ showed increased height compared to those grown under natural light (Figure 1A,B,E,F). PAC treatment suppressed endogenous GA_3_ synthesis, resulting in reduced plant height in wild type and mutants compared to those grown under natural light. Notably, *OsCSN1*-*GFP*-OE experienced a significant reduction in height (Figure 1A,B,G,H). Therefore, OsCSN1 may be a positive regulator of far-red light. The N-terminal of OsCSN1 may be an important key domain in the regulation of far-red light.

### 2.2. OsCSN1 Affects the Length of the Coleoptile by Far-Red Light Through the N-Terminal

Under far-red light, the coleoptiles of the *oscsn1*-*2*, *OsCSN1DN102*-*GFP*-OE, and *OsCSN1*-*GFP*-OE increased significantly compared with those of the wild type, whereas the coleoptile length of *OsCSN1DN32*-*GFP*-OE was inhibited (Figure 1C,D). Exogenous gibberellin (GA_3_) treatment further promoted coleoptile elongation in wild type and mutants under far-red light. Notably, *oscsn1*-*1* and *OsCSN1DN32*-*GFP*-OE exhibited significantly longer coleoptiles compared to those under far-red light (Figure 1E,F). In contrast, treatment with the exogenous hormone PAC inhibited coleoptile growth in wild type and mutants, with the most significant inhibition observed in *oscsn1*-*1* (Figure 1G,H).

Compared with the coleoptile of wild type and mutants under natural light, the coleoptile of the *OsCSN1DN32*-*GFP*-OE under far-red light did not significantly change compared with that of the wild type, while the coleoptile of the wild type and the remaining mutants increased (Figure 1A–D). After the addition of the exogenous hormone GA_3_, the coleoptile of the wild type and mutants increased compared to that under natural light, and the coleoptile of the *oscsn1*-*1* significantly increased compared to that of those under natural light (Figure 1A,B,E,F), as they are sensitive to the effect of the exogenous hormone GA_3_. After the addition of the exogenous hormone PAC, the growth of the coleoptiles of the wild type and mutants were inhibited (Figure 1A,B,G,H).

The results showed that, because the far-red light elongated the coleoptiles of the mutants, the coleoptiles of the plants under the combined treatment of the exogenous hormone GA_3_ and the far-red light also grew, while the addition of exogenous hormone PAC inhibited the synthesis of endogenous gibberellin in the plants and the coleoptile elongation of the plants showed a significant inhibitory effect. The coleoptile of the *OsCSN1*-reduced mutant and *OsCSN1DN102*-*GFP*-OE changed significantly under far-red light and hormones. Therefore, it was speculated that the N-terminal deletion of OsCSN1 might affect coleoptile length under far-red light.

### 2.3. OsCSN1 Inhibits Root Length Elongation Mediated by Far-Red Light Through the N-Terminal

The root length of the mutants decreased under far-red light and the root lengths of the *oscsn1*-*1* and *OsCSN1DN32*-*GFP*-OE were significantly inhibited, while the root length of the wild type under far-red light was significantly increased (Figure 1C,D). The root length of the *OsCSN1DN102*-*GFP*-OE was significantly inhibited after the addition of the exogenous hormone GA_3_ (Figure 1E,F). After the addition of the exogenous hormone PAC, the root length of the *oscsn1*-*1* increased and the root length of the *OsCSN1*-*GFP*-OE was significantly inhibited under far-red light (Figure 1G,H).

The root length of the mutants under far-red light was shorter than that under natural light (Figure 1A–D). After the addition of the exogenous hormone GA_3_, the root length of wild type and mutants were inhibited compared with natural light (Figure 1A,B,E,F). After the addition of the exogenous hormone PAC, the root length of the wild type and mutants were inhibited compared to that under natural light, and the root length of *OsCSN1*-*GFP*-OE was significantly inhibited (Figure 1A,B,G,H).

The results show that far-red light-mediated inhibition of root length is associated with OsCSN1, particularly its N-terminal domain. Exogenous GA_3_ could not rescue this inhibition, indicating that OsCSN1 acts as an inhibitor of root growth under far-red light, with its primary regulatory domain located at the N-terminal.

### 2.4. Far-Red Light Signals and Hormone Signals Affect the Expression of Proteins and Genes in Mutants

**Figure 2:**
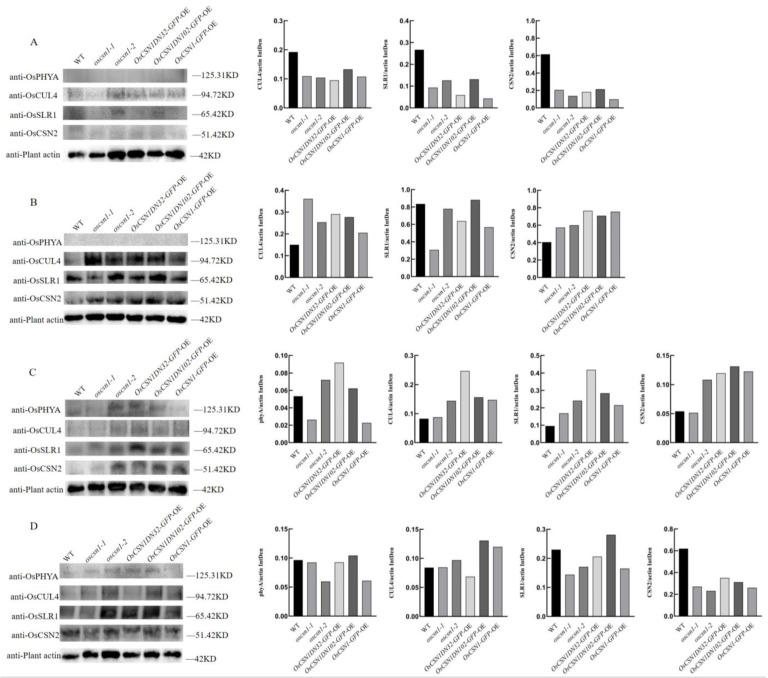
OsCSN1 regulates seedling growth under far-red light by regulating the GA pathway. Protein expression of wild type and *OsCSN1* mutants grown 9d under different conditions. Detection of OsSLR1, OsCSN2, OsCUL4, and OsphyA protein levels in samples lines using a western blot. (**A**) Under natural light. (**B**) Under far-red light. (**C**) Under far-red and GA_3_ co-processed. (**D**) Under far-red and PAC co-processed.

**Figure 3:**
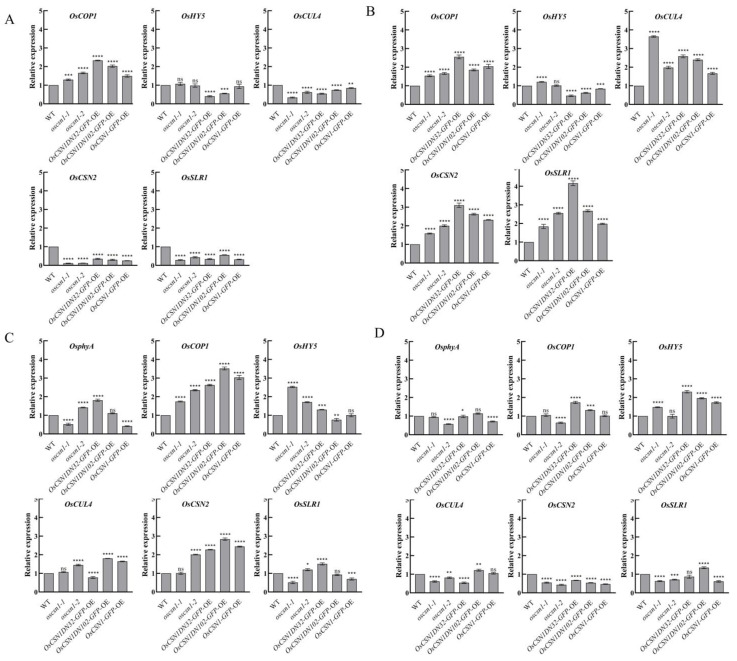
Changes and interactions of OsCSN1-related genes during seedling growth under far-red light. The average values (±SD) from three biological repeats are shown. For each gene, different letters indicate significant differences in expression according to Duncan’s multiple range test, * *p* < 0.05; ** *p* < 0.01; *** *p* < 0.001; **** *p* < 0.0001; ns stands for no significant difference. Expression of genes associated with wild type and *OsCSN1* mutants grown for 9d under different conditions of treatment at the mRNA level. Expression of *OsSLR1*, *OsCSN2*, *OsCUL4*, *OsCOP1*, *OsHY5*, and *OsphyA* genes in the sample lines was examined using qPCR. (**A**) Under natural light. (**B**) Under far-red light. (**C**) Under far-red light and GA_3_ co-processed. (**D**) Under far-red light and PAC co-processed.

Under far-red light, the expression level of the OsSLR1 protein in *oscsn1*-*1* was significantly upregulated. On the other hand, the expression level of the OsSLR1 protein in the *oscsn1*-*2* and *OsCSN1DN102*-*GFP*-OE was significantly upregulated compared with that in the control group under natural light (Figure 2A,B). The expression level of the *OsSLR1* gene was significantly upregulated in the *oscsn1*-*2* (Figure 3A,B). After the addition of the exogenous hormone GA_3_, the OsSLR1 protein expression level in the wild type, *oscsn1*-*2,* and *OsCSN1DN102*-*GFP*-OE was significantly upregulated (Figure 2A,C). After the addition of the exogenous hormone PAC, the expression level of the OsSLR1 protein in the wild type, *oscsn1*-*2*, and *OsCSN1DN102*-*GFP*-OE was significantly downregulated (Figure 2A,D), while the expression level of the *OsSLR1* gene in the *oscsn1*-*2* was significantly downregulated (Figure 3A,D).

Under far-red light, the OsCSN2 expression levels of the *OsCSN1DN32*-*GFP*-OE and *OsCSN1*-*GFP*-OE were significantly upregulated (Figure 2A,B and Figure 3A,B). After the addition of the exogenous hormone GA_3_, the expression level of the OsCSN2 in the *OsCSN1DN32*-*GFP*-OE and *OsCSN1*-*GFP*-OE was significantly downregulated (Figure 2A,C). Similarly, the expression level of the *OsCSN2* gene was significantly downregulated in the *OsCSN1DN32*-*GFP*-OE (Figure 3A,C). After the addition of the exogenous hormone PAC, the OsCSN2 expression level was upregulated in wild type, while the OsCSN2 expression levels in the mutants were downregulated. In addition, the OsCSN2 expression level was significantly downregulated in the *OsCSN1DN32*-*GFP*-OE and *OsCSN1*-*GFP*-OE (Figure 2A,D) and the expression level of the *OsCSN2* gene was significantly downregulated in the mutants (Figure 3A,D).

Under far-red light, the OsCUL4 expression levels were upregulated in the mutants and the expression level in the *oscsn1*-*1* plants was significant upregulated (Figure 2A,B and Figure 3A,B). After the addition of the exogenous hormone GA_3_, the OsCUL4 expression level in *oscsn1*-*1* was significant upregulated (Figure 2A,C and Figure 3A,C). After the addition of the exogenous hormone PAC, the OsCUL4 protein expression level in *oscsn1*-*1* and *OsCSN1DN32*-*GFP*-OE was significantly downregulated (Figure 2A,D and Figure 3A,D).

Under far-red light, neither the wild type’s nor the mutants’ OsphyA was expressed (Figure 2A,B). After the addition of the exogenous hormone GA_3_, the OsphyA and *OsphyA* gene expression levels in the wild type and mutants were upregulated compared to those in the far-red light, with the *OsCSNDN32*-*GFP*-OE showing a significant upregulation in the OsphyA protein and *OsphyA* gene expression levels (Figure 2A,C and Figure 3A,C). After the addition of the exogenous hormone PAC, the expression of the OsphyA protein and *OsphyA* gene in the wild type and mutants was upregulated compared with that under far-red light and the expression of OsphyA protein and *OsphyA* gene in *OsCSN1DN102*-*GFP*-OE was upregulated significantly (Figure 2A,D and Figure 3A,D).

Under far-red light, the expression level of the *OsCOP1* gene was downregulated compared with that under natural light, while the expression level of the *OsHY5* gene was upregulated (Figure 3A,B). The expression level of the *OsCOP1* gene was significantly downregulated in the *OsCSN1DN32*-*GFP*-OE and *OsCSN1DN102*-*GFP*-OE (Figure 3A,B). After the addition of the exogenous hormones GA_3_ and PAC, the gene expression of *OsCOP1* and *OsHY5* was upregulated compared with that in the far-red light. The gene expression levels of *OsCOP1* and *OsHY5* in the *OsCSN1DN32*-*GFP*-OE were significantly upregulated (Figure 3A,C,D).

## 3. Discussion

### 3.1. OsCSN1 Localization Affects Rice Seedling Growth

Under far-red light, GFP expression was absent in the leaf tips and coleoptile veins of the *OsCSN1DN32*-*GFP*-OE and *OsCSN1DN102*-*GFP*-OE (Figure S1B). In contrast, the *OsCSN1*-*GFP*-OE exhibited extensive GFP expression in both leaves and coleoptiles, with limited expression in roots (Figure S1B). Overexpression of OsCSN1 inhibited both the aboveground and underground parts of the *OsCSN1*-*GFP*-OE (Figure 1A,B). In contrast, the aboveground height of the *OsCSN1DN32*-*GFP*-OE was significantly inhibited, the plant height of the *OsCSN1DN102*-*GFP*-OE was inhibited, and the coleoptiles were elongated under far-red light (Figure 1A,B). These observations suggest that deletions of 32 or 102 amino acids in the N-terminal region of OsCSN1 may induce conformational changes that impair far-red light signal reception, leading to distinct phenotypic differences.

Under the combined regulation of far-red light and exogenous GA_3_, OsCSN1-GFP-OE displayed abundant GFP expression in both leaves and roots (Figure S1C). In the *OsCSN1DN32*-*GFP*-OE, the addition of the exogenous hormone GA_3_ promoted the elongation of the aboveground and underground parts. However, in *OsCSN1DN102*-*GFP*-OE, exogenous GA_3_ reduced the overall plant height while promoting coleoptile elongation (Figure 1A,C). For *OsCSN1*-*GFP*-OE, GA_3_ application stimulated shoot growth but suppressed root growth. Furthermore, in *OsCSN1*-*GFP*-OE roots, GFP protein expression was significantly reduced, correlating with inhibited root elongation and protein expression.

Under the synergistic regulation of far-red light and exogenous hormone PAC, the *OsCSN1DN32*-*GFP*-OE and *OsCSN1DN102*-*GFP*-OE produced GFP proteins in the leaf tips, but a small amount of GFP was fused in the roots of the *OsCSN1DN32*-*GFP*-OE. Protein expression analysis revealed that there was a small amount of GFP protein in the coleoptiles of the *OsCSN1DN102*-*GFP*-OE (Figure S1D). The *OsCSN1*-*GFP*-OE expressed a large amount of the GFP protein in the leaves, coleoptiles, and roots (Figure S1D). The aboveground and underground parts of the *OsCSN1DN32*-*GFP*-OE, *OsCSN1DN102*-*GFP*-OE, and *OsCSN1*-*GFP*-OE all exhibited inhibition (Figure 1A,D). These results suggest that far-red light and GA signaling pathways jointly regulate seedling growth via OsCSN1, with far-red light signaling predominantly influencing plant height, while GA signaling has a stronger effect on coleoptile and root elongation. The phenotypic differences between the aerial and underground parts may result from differential signal reception, warranting further investigation into the exact regulatory mechanisms.

### 3.2. OsCSN1 Is Involved in the Regulation of Rice Plant Phenotype Through N-Terminal Under Far-Red Light and There Are Different Sensitivities and Synergistic Effects with Hormone Signals

The COP9 signalosome (CSN) is a highly conserved multi-subunit protein complex, with CSN1 being its largest and most conserved subunit. The N-terminal region of CSN1 plays a pivotal role in photomorphogenesis and seedling development, making it critical for plant survival [11]. Structural analysis of OsCSN1 revealed that the deletion of 32 amino acids from the N-terminal did not significantly affect its tertiary structure. However, the deletion of 102 amino acids from the N-terminal led to a substantial conformational change [32]. This study demonstrated that both *OsCSN1DN32* and *OsCSN1DN102* mutants exhibited normal growth during the seedling stage under far-red light but displayed varying degrees of phenotypic alterations. These findings highlight the N-terminal region of OsCSN1 as a critical domain for regulating rice seedling development under far-red light.

OsCSN1 is a positive regulator of seedling growth regulated by far-red light and its main domain is located at the N-terminal. The OsCSN1 N-terminal deletion mutants are sensitive to far-red light and the effect of far-red light is more prominent. After the addition of exogenous hormones and far-red light, OsCSN1 was found to have a deletion of 32 amino acids at the N-terminal. The far-red light signal and hormone signal responses showed significant sensitivity. The overexpression of OsCSN1 allowed far-red light and hormone signals to regulate plant height increases in rice seedlings through OsCSN1. Plants with reduced OsCSN1 expression exhibit coleoptile growth under far-red light and the coleoptile elongation in *OsCSN1* mutants is more sensitive to hormonal signals (Figure 1).

Far-red light influenced the root length of the OsCSN1 N-terminal deletion mutants, with the root showing heightened sensitivity to far-red light signals. The addition of the hormones GA_3_ and PAC inhibited the root length of the mutants. Notably, GA_3_ did not mitigate the detrimental effects of far-red light on the root (Figure 1).

### 3.3. OsCSN1 Responds to Far-Red Light by Regulating the SLR1 Factor in the GA Pathway

Hormone signaling plays a crucial role in regulating seed germination and seedling growth. Under far-red light, OsSLR1 protein and gene expression was upregulated in both wild type and mutants (Figure 2A,B and Figure 3A,B). However, when far-red light was combined with exogenous GA_3_ or PAC, both OsSLR1 protein and gene expression were downregulated in the mutants (Figure 2A,C,D and Figure 3A,C,D). Under far-red light, the plant height and root length were inhibited in *OsCSN1* mutants (Figure 1A,B). After the addition of the exogenous hormone GA_3_, the plant height of the *OsCSN1DN102*-*GFP*-OE was significantly inhibited (Figure 1A,C). Similarly, the addition of PAC resulted in inhibited plant height and root length in the mutants (Figure 1A,D).

OsCSN1 exhibited sensitivity to far-red light and GA signals in the aboveground and underground parts of the N-terminal deletion mutants. These findings suggest that far-red light may regulate OsSLR1 expression through OsCSN1, thereby influencing GA signaling pathways and modulating rice seedling growth. The primary functional domain of OsCSN1 resides in its N-terminal region.

### 3.4. OsCSN1 Senses Far-Red Light Signals Through phyA to Regulate Seedling Growth

Light plays a critical role in plant growth and development. Under far-red light, the expression levels of *OsCOP1*, *OsHY5*, *OsCSN2*, and *OsCUL4* in the *OsCSN1DN32*-*GFP*-OE were upregulated (Figure 3A,B). However, when far-red light was combined with exogenous hormones, *OsCSN2* and *OsCUL4* gene expression was significantly downregulated, while *OsHY5* gene expression was upregulated (Figure 3A,B). Additionally, when far-red light and the exogenous hormone GA_3_ were applied together, the expression of the *OsCOP1* gene was significantly increased in both the *OsCSN1DN32*-*GFP*-OE and *OsCSN1DN102*-*GFP*-OE (Figure 3A,C).

PhyA, which shifts from its active Pfr form to the inactive Pr form under far-red light, was not expressed in the mutants under far-red light exposure (Figure 2A,B and Figure 3A,B). Under the synergistic regulation of far-red light and an exogenous hormone, the expression of the OsphyA and *OsphyA* gene was upregulated in the mutants. Under the synergistic regulation of far-red light and exogenous hormone, the growth of the underground part of the mutants was inhibited and the expression of *OsphyA* increased. The OsCSN1 deletion N-terminal 32 amino acid (*OsCSN1DN32*-*GFP*-OE) mutant also showed inhibition due to the increased expression of *OsphyA*. This finding is consistent with the finding that rice senses far-red light signals through phyA to inhibit coleoptile and root elongation [34,35]. Under far-red light, the phytochrome Pr and Pfr types switch and thus regulate the growth and development of rice plants through the metabolism of various hormones, affecting the growth of rice plants [36].

Previous studies have shown that after receiving a far-red light signal, phyA binds to FHY1/FHL, is transported into the nucleus, and is converted to the inactive Pr form [36,37]. phyA-Pfr inhibits the function of COP1, thereby promoting the accumulation of HY5. HY5 inhibits the transcriptional activation of FHY1/FHL via FHY3/FAR1 [25] and negatively regulates the entry of phyA into the nucleus. Moreover, phyA can also inhibit the transcription of FHY3/FAR1, thereby inhibiting FHY1/FHL in the entry and exit cores [38]. Therefore, phyA may sense the far-red light signal and convert it to the inactive Pr form to increase the biological activity of GA, thereby inhibiting the expression of SLR1.

**Figure 4:**
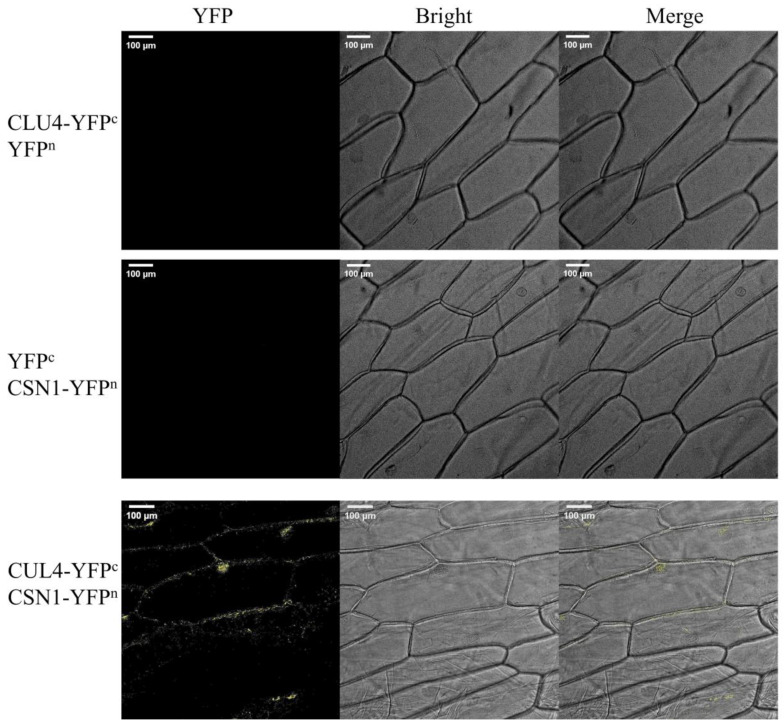
The interaction between OsCSN1 and OsCUL4 was detected by bimolecular fluorescence complementation. “CUL4-YFP^c^ + YFP^n^” and “YFP^c^ + CSN1-YFP^n^” were single-molecule control groups and no fluorescence was detected. “CUL4-YFP^c^ + CSN1-YFP^n^” was the experimental group. Under the conditions of the normal negative and positive control, fluorescence could be detected in the experimental group, indicating that the two proteins could interact with each other. Bar = 100 μm.

**Figure 5:**
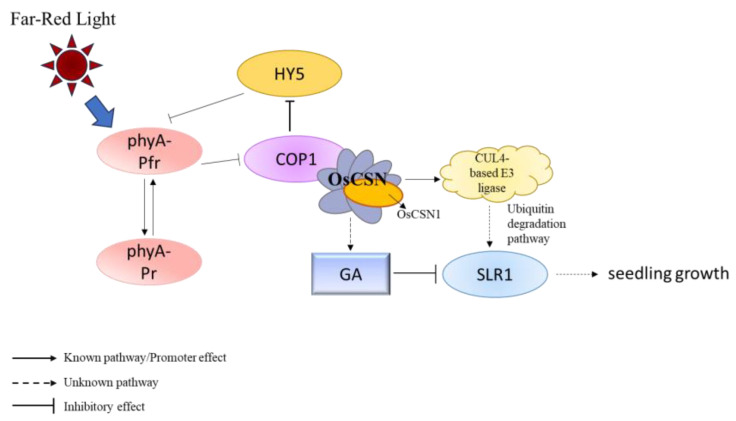
Presumed far-red light mediates the signaling pathway of OsCSN1 in regulating seedlings.

Far-red light also upregulated the expression of OsCSN2 and OsCUL4. It is proposed that OsCSN1, upon sensing far-red light, forms part of the COP9 complex, interacting with COP1 to regulate OsSLR1 degradation via OsCUL4-mediated ubiquitination in the CULLIN family. The results showed that the interaction between OsCUL4 and OsCSN1 was proved by a yeast two-hybrid experiment [32] and BiFC experiment (Figure 4). Far-red light signal activates phyA and converts it into the inactive Pr form. After sensing far-red light, OsCSN1 interacts with COP1 by forming a COP9 complex and then regulates the degradation of OsSLR1 through OsCUL4-mediated ubiquitination, while OsSLR1 is a negative regulator of GA signaling. This process affects the growth and development of rice seedlings, especially the elongation of seedling height, by regulating the GA signaling pathway (Figure 5).

### 3.5. Limitations and Potential of Far-Red Light in Regulating Rice Growth

Previous studies have shown that, while far-red light can promote growth, promote flowering and the chlorophyll fluorescence response, etc. [39], far-red light does have some limitations in regulating rice growth and development which may affect its effectiveness as an independent agricultural management tool. Plant growth requires a variety of light qualities, including blue light, red light, and far-red light. The application of a single type of far-red light may lead to spectral imbalance, thus affecting the overall health and productivity of plants. For example, excessive far-red light may lead to the overgrowth of plants, reduced mechanical strength, and reduced photosynthetic efficiency of leaves. Under natural conditions, the light conditions are complex and changeable and a with a single far-red light treatment it is difficult to achieve the desired effect stably. Environmental factors such as light intensity, photoperiod, and temperature may affect the interaction between far-red light and hormone pathways. Although far-red light has its unique advantages in the regulation of rice growth and development, it has many limitations as the only means of regulation. In order to overcome these problems, it is usually recommended to adopt a comprehensive light management strategy, combined with a variety of light quality and other agronomic measures, to ensure that rice can obtain balanced and suitable light conditions throughout the growth cycle, taking into account economic benefits and ecological adaptability. Although far-red light has some limitations in regulating the growth and development of rice, it also has unique application potential which can bring significant benefits to agricultural production. LED lights or other artificial light sources have been used to supplement or adjust the proportion of far-red light to optimize the growth environment of rice seedlings. By controlling the daily light duration and intensity, the diurnal variation found under natural conditions is simulated, especially the low-angle sunlight in the morning and evening, which is rich in far-red light. This helps to enhance the shade avoidance response of plants. Based on the research results of key regulatory factors such as OsCSN1, new varieties that can respond more effectively to far-red light signals were developed through gene editing, providing innovative solutions for modern agricultural practice.

In general, OsCSN1 plays a key role in regulating plant growth and development in response to far-red light. Future studies should continue to explore the specific mechanisms of far-red light on the regulation of other subunits of the COP9 signaling complex in rice and how to improve the productivity and adaptability of rice through these mechanisms. This is not only of great significance to basic research, but also brings potential benefits to the practice of agricultural production.

## 4. Materials and Methods

### 4.1. Plant Material and Culture Conditions

The construction method and source of the mutants were provided by our laboratory [32,33,40]. The seeds of the wild type (*Oryza sativa* subsp. *japonica*), *OsCSN1* knockout (*oscsn1*-*1*) mutant, *OsCSN1*-reduced (*oscsn1*-*2*) mutant, *OsCSN1* overexpression (*OsCSN1*-*GFP*-OE) mutant, and *OsCSN1* deletion N-terminal (*OsCSN1DN32*-*GFP*-OE and *OsCSN1DN102*-*GFP*-OE) mutant plants were divided into four groups. Then, 0.8% (*w*/*v*) agar medium was prepared, 10 mM GA_3_ and 100 mM endogenous gibberellin inhibitor paclobutrazol (PAC) were added to the medium, respectively, and the 5 seeds of the wild type and mutants were sown on the medium. The seeds in the experimental group underwent light irradiation with LED-FR (10 μmol m^−2^s^−1^) (Shenzhen Lvheng Lighting Technology Co., Ltd., Shenzhen, China) for 24 h in a light incubator. Another group of seeds, as the control group, was sown on 0.8% (*w*/*v*) agar medium and positioned in a light incubator (Ningbo Jiangnan Instrument Factory, Ningbo, China) with a light intensity of 203 μmol m^−2^s^−1^ in a 12 L:12 D light cycle. The temperature during incubation was maintained at 28 ± 1 °C and the relative humidity during incubation was maintained at 95%.

### 4.2. Phenotypic Analysis of Rice Seedlings

Seeds of the wild type and mutants were cultured under the above conditions for 9 days. Phenotypic data were measured, including plant height, coleoptile length, and root length. All measurements for each group were repeated 5 times (n = 5).

### 4.3. Protein Extraction and Western Blot Analysis

A total of 0.5 g sample was ground in liquid nitrogen, mixed with Extraction Buffer (PI, PMSF, and DTT were added to the plant RIPA lysate in proportion), And vortexed and centrifuged at 4 °C for 10 min. The supernatant was taken and 5 × SDS-PAGE sample buffer was added and mixed. After boiling for 10 min, western blot testing was performed.

For western blotting, the modified Mahmood method was used [41]. Each sample was separated via 10% SDS-PAGE. The SDS-PAGE gels were subsequently transferred to polyvinylidene fluoride (PVDF) membranes using the Bio-Rad Trans-Blot Turbo blotting system. The proteins on the PVDF membranes were blocked with 4% skim milk. After incubation with the appropriate antibody, the target protein bands were visualized via chemiluminescence. The rabbit polyclonal antibodies used in this experiment included OsCUL4, OsCSN2, OsCSN5, OsSLR1, and OsphyA and an anti-plant actin mouse polyclonal antibody. The following secondary antibodies were used: goat anti-rabbit and goat anti-mouse IgG secondary antibody (HRP). The antibodies used in this study were purchased from Wuhan ABclonal Biotechnology Co., Ltd. (Wuhan, China).

### 4.4. Total RNA Extraction and Real-Time Quantitative Polymerase Chain Reaction

Total RNA was extracted from the seedlings using a Spectrum Plant Total RNA Kit (Sigma-Aldrich, Darmstadt, Germany). About 0.5 mg of cDNA was synthesized using StarScript II first-strand cDNA synthesis mix (GenStar, Beijing, China). Rapid quantitative polymerase chain reaction (PCR) was completed using a mixture of 2x RealStar Green Fast and ROX (GenStar, Beijing, China) on a StepOnePlusTM real-time PCR device (Thermo Fisher Scientific, Applied Biosystems, Waltham, MA, USA). The blank control gene *GAPDH* (JN848809) was used to quantify the relative mRNA levels calculated by averaging three replicates. The primers used in the experiment are shown in Table 1.

### 4.5. Spatiotemporal Positioning

Three proteins, OsCSN1DN32, OsCSN1DN102, and OsCSN1, were constructed as fusion proteins with green fluorescent protein (GFP) and were effectively expressed; subsequently, the proteins were observed and analyzed via laser scanning confocal microscopy. The GFP was observed in the leaves, coleoptiles, and roots of OsCSN1-overexpressing plants that grew for 9 days. Images were obtained using a Leica TCS SP8 CARS confocal microscope (Leica, Wetzlar, Germany) with a 10 × 0.40 eyepiece and 20× and observed with an objective lens of 0.70. The selected positioning point was excited with a 488 nm laser and we detected GFP fluorescence with a 500–530 nm band.

### 4.6. Bimolecular Fluorescence Complementation

PCR amplification was performed by using designed primers and the target gene as templates. The primers used in the experiment are shown in Table 2. The vectors pSAT4A-cEYFP-N1 and pSAT4A-nEYFP-N1 were linearized by *EcoR*I (Thermo Fisher Scientific, Waltham, MA, USA). OsCUL4-pSAT4A-cEYFP-N1 and OsCSN1-pSAT4A-nEYFP-N1 were constructed using a ClonExpress II One Step Cloning Kit-C112 (Vazyme, Nanjing, China). They were transferred into onion epidermal cells by an Agrobacterium-mediated method and cultured in the dark for 24 h for observation. Images were obtained using a Leica TCS SP8 CARS confocal microscope (Leica, Wetzlar, Germany) with a 10× eyepiece and 0.70 objective lens for observation. YFP fluorescence with an excitation wavelength of 514 nm and emission wavelength of 527 nm was detected.

### 4.7. Data Processing and Analysis

The data are expressed as the standard error of the mean for the control group and the experimental group. *p* ≤ 0.05 indicates statistical significance. ImageJ (1.52V) software was used to analyze the results of western blot. The experimental data were analyzed using Excel 2019 and SPSS 28.0 and the graphs were drawn using GraphPad Prism 8.

## 5. Conclusions

This study shows that OsCSN1 plays a crucial role in regulating the growth of rice seedlings under far-red light. The N-terminal region of OsCSN1 is essential for its function and this domain is an important site in the response to far-red light and hormone signals. When a far-red light signal is perceived, phyA is converted to its inactive Pr form, which promotes COP1 and inhibits HY5 accumulation. After sensing the far-red light signal, OsCSN1 forms a COP9 signaling complex and interacts with COP1, then regulates the degradation of the key regulator OsSLR1 in the GA signaling pathway through OsCUL4-mediated ubiquitination. This process affects aboveground and underground growth. Far-red light mainly regulates stem elongation and GA signals affect coleoptile elongation more strongly. In addition, the interaction between OsCSN1 and OsCUL4 in the far-red light signaling pathway highlights its central role in coordinating light and hormone signals to regulate rice growth. These findings highlight the importance of the N-terminus of OsCSN1 in mediating complex interactions between light and hormone regulation during plant development. Based on this mechanism, new rice varieties that can use far-red light signals more efficiently can be cultivated in the future, thereby improving the growth rate, adaptability, and productivity of rice. By optimizing the sensing mechanism of crop light signals, this approach can not only improve the yield of crops but also provide a more sustainable development plan for agricultural production.

## Figures and Tables

**Table 1:** All the primers that were used for qRT-PCR.

Primer	Sequence (5′-3′)
GAPDHF	AAGCCAGCATCCTATGATCAGATT
GAPDHR	CGTAACCCAGAATACCCTTGAGTTT
Dye-SLR1F	CATGCTTTCCGAGCTCAACG
Dye-SLR1R	TGACAGTGGACGAGGTGGAA
Dye-CUL4F	AGGACAGACAGTATCAGGTGGATGC
Dye-CUL4R	TCCGATGGCTTGATTGGGAACTTG
Dye-COP1F	CATCTCAGCCACAAGAGCGACTG
Dye-COP1R	GGTCTATCGGTGATGCTGTCTTCG
Dye-CSN2F	GAGCAGCTCTTGGTCTCACTCATTC
Dye-CSN2R	CGACCTGTCACCACGTTCTAGTAAC
Dye-phyAF	GATGGTGCTCTGAGTGGAATGC
Dye-phyAR	ACAGGAGGCGTTGGTGCTATC
Dye-HY5F	AGGTGAAGGTGAAGGACTTGGAG
Dye-HY5R	GAGCATCTGGTTCTCATTCTGTAGG

**Table 2:** All the primers that were used to BiFC.

Primer	Sequence (5′-3′)
CSN1-c/nEYFP-F	GATCTCGAGCTCAAGCTTCGAATTCATGGACGTCGAGGGCGAGGTCCCGG
CSN1-nEYFP-R	TCGCCCTTGCTCACCATCAGGATCCCATCTTCCTTTGTCCAGCTCTTTGG
CUL4-c/nEYFP-F	GATCTCGAGCTCAAGCTTCGAATTCATGCACAAAAACTAAGCTTC
CUL4-cEYFP-R	GCGAGCTGCACGCTGCCCAGGATCCAGCCAGGTAATTGTAGATCT

## Data Availability

The data presented in this study are available in the article.

## Supplementary Materials

The following supporting information can be downloaded at: https://www.mdpi.com/article/10.3390/ijms26020522/s1.
