# Supplementary material for: Functional Characterization of OsCSN1 in the Agronomic Trait Control of Rice Seedlings Under Far-Red Light
Source: Int J Mol Sci. 2025 Jan 9;26(2):522. doi: 10.3390/ijms26020522 (PMC11765280; doi:10.3390/ijms26020522)
Supplement: Supplementary file 1 [file ijms-26-00522-s001.zip › Supplementary Information.pdf]

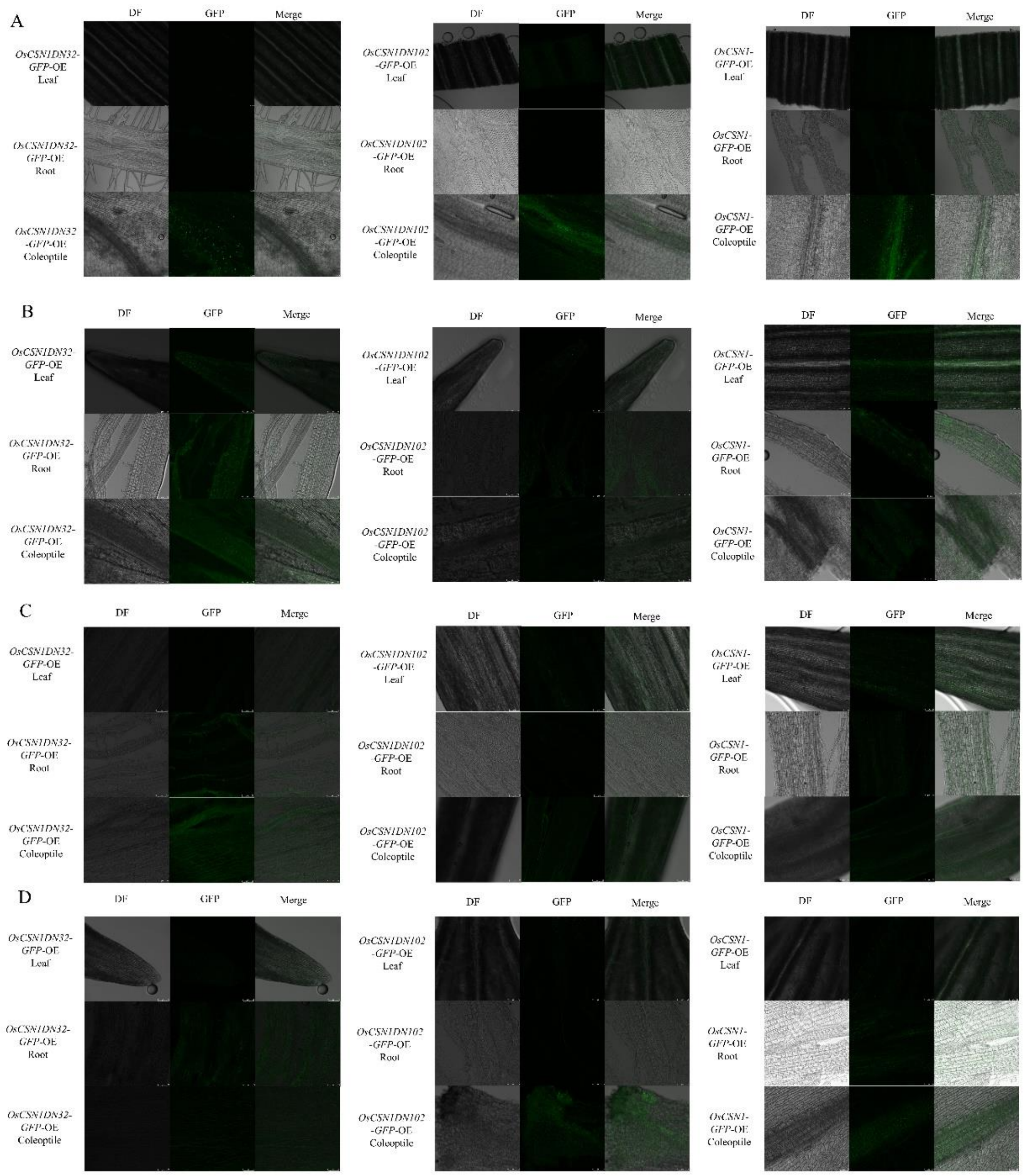

Fig.S1 The expression of OsCSN1 affects its spatial and temporal localization in rice.

(A) The expression of OsCSN1, OsCSN1DN32 and OsCSN1DN102 in leaf, coleoptile and root under light.

(B) The expression of OsCSN1, OsCSN1DN32 and OsCSN1DN102 in leaf, coleoptile and root under far-red light.

(C) The expression of OsCSN1, OsCSN1DN32 and OsCSN1DN102 in leaf, coleoptile and root under far-red light and GA_3 co-processed.

(D) The expression of OsCSN1, OsCSN1DN32 and OsCSN1DN102 in leaf, coleoptile and root under far-red and PAC co-processed.

Bar=25 μm.
